# Current Strategies for Selenium and Iodine Biofortification in Crop Plants

**DOI:** 10.3390/nu14224717

**Published:** 2022-11-08

**Authors:** Eva Duborská, Martin Šebesta, Michaela Matulová, Ondřej Zvěřina, Martin Urík

**Affiliations:** 1 Institute of Laboratory Research on Geomaterials, Faculty of Natural Sciences, Comenius University in Bratislava, Mlynská dolina, Ilkovičova 6, 84215 Bratislava, Slovakia; 2 Department of Public Health, Faculty of Medicine, Masaryk University, Kamenice 5, 62500 Brno, Czech Republic

**Keywords:** selenium, iodine, deficiency, biofortification, nutrition, crops

## Abstract

Selenium and iodine are essential trace elements for both humans and animals. Among other things, they have an essential role in thyroid function and the production of important hormones by the thyroid gland. Unfortunately, in many areas, soils are deficient in selenium and iodine, and their amount is insufficient to produce crops with adequate contents to cover the recommended daily intake; thus, deficiencies have an endemic character. With the introduction of iodized table salt in the food industry, the thyroid status of the population has improved, but several areas remain iodine deficient. Furthermore, due to the strong relationship between iodine and selenium in metabolic processes, selenium deficiency often compromises the desired positive impact of salt iodization efforts. Therefore, a considerable number of studies have looked for alternative methods for the simultaneous supplementation of selenium and iodine in foodstuff. In most cases, the subject of these studies is crops; recently, meat has also been a subject of interest. This paper reviews the most recent strategies in agriculture to fortify selenium and iodine in crop plants, their effect on the quality of the plant species used, and the potential impact of food processing on their stability in fortified crops.

## 1. Introduction

Iodine and selenium are both essential for humans and animals; among other functions, they play an important role in thyroid gland function and thyroid hormone synthesis and metabolism [1]. The Recommended Daily Allowance (RDA) for iodine and selenium depends on age and gender and amounts to 90–250 µg and 60–70 µg per day, respectively [2,3]. A lack of selenium in people’s diets results in disruption of the antioxidant cell system, which can lead to dysfunction of the brain, cardiovascular system, liver, and muscles. Previous studies have also proven higher mortality caused by hypertension and ischemic and arthritis diseases connected to insufficient selenium intake. Impairment of glutathione peroxidase activity due to a lack of selenium results in impairment of immunity and the emergence of many diseases, such as cystic fibrosis and multiple sclerosis [4]. Insufficient I intake causes prenatal mortality, cognitive impairment, hypo- and hyperthyroidism, and increased susceptibility of the thyroid gland to nuclear radiation [5,6].

Generally, both minerals enter the food chain when plants take them up from the soil and accumulate them in edible parts. Their poor transport in plant tissues and low accumulation rates due to their limited bioavailability from soils has resulted in a worldwide deficit of I and Se in the diets of humans and animals, especially in areas where soils are also I and Se deficient [7,8]. Consequently, biofortification is an effective crop-based approach to dealing with the mineral malnutrition problem, so-called hidden hunger, by enriching crops and food products with bioavailable micronutrients [9].

The supplementation of staple crops by means of fertilizers with deficient minerals is an effective and readily controllable way of increasing their average daily intake [10]. Understanding the mechanism of the soil-plant nutrient system is essential for successful biofortification methods [9]. Before agronomic biofortification, we need to understand which speciation of the desired micronutrient is found in soils and which form can be taken up by plants. Minerals are often unevenly distributed among different plant parts, though for a successful biofortification process, plants should accumulate minerals in their edible parts [11]. There are three main approaches to biofortification. While the (i) agronomic approach focuses on the application of fertilizers and mobilization of minerals, (ii) conventional plant breeding and (iii) genetic engineering approaches aim at improving plant varieties in order to achieve a higher capacity to accumulate micronutrients in the edible plant tissues and to increase their bioavailability through a higher concentration of promoter substances and a lower concentration of antinutrients [10]. The effectiveness of biofortification programs relies on their acceptance by farmers and consumers and future policy interventions; thus, the regular consumption of biofortified varieties is a key factor that decides the impact of biofortification programs [9].

## 2. Health Aspects

### 2.1. Selenium in the Human Body

The continuous intake of low-selenium content food causes complicated health-related problems such as epilepsy, oxidative stress-related conditions, infertility, and immune deficiency [12]. Selenium deficiency plays a role in numerous metalloenzyme systems in the body and is most notably known for its role in the activity of the antioxidant glutathione peroxidase. The selenium-dependent enzyme glutathione peroxidase protects cell membranes from peroxidative damage [12] and is recognized as one of the most powerful mediators of oxidative stress in mammals [13]. Se also reacts with heavy metals (such as Hg, Cd, As, and Pb) forming insoluble selenides, which prevents poisoning [14].

Selenium deficiency disorders often have an endemic character in areas where soils are Se deficient. The selenium concentration in human blood is approximately 27 μg∙L^−1^ in geographical areas with low selenium intake. An intake of 90 μg of selenium per day would correspond to around 110 μg∙L^−1^ [15]. Keshan disease is a type of cardiomyopathy associated with heart failure, cardiac enlargement, and abnormalities in electrocardiograms and heart rhythms first described in Keshan County, a region of China with endemic Se deficiency. In such areas, Kashin–Beck disease was also described, this causing the disabling deformity of bones and joints [12]. Selenium also affects the immune system. It has been shown that in host individuals with low serum selenium content, harmless viruses are turned into virulent pathogens. Selenium is also a crucial nutrient in HIV-infected patients and those struggling with cancer, COVID-19, and Hepatitis B and C, since Se has been proven to activate the production of T cells and stimulate the production of antibodies [12,16,17,18]. It is assumed that selenium is also beneficial in states of depression and Alzheimer’s disease and is essential for the reproductive system [12,19,20].

Despite its essentiality, selenium is toxic in high concentrations. Selenium has one of the narrowest ranges between dietary deficiency (<40 μg∙day^−1^) and toxic levels (>400 μg∙day^−1^) for humans [21]. Moreover, selenite (IV) is four times more toxic than selenate (VI) [22]. Toxic effects appear when serum selenium concentrations span up to 30,000 μg∙L^−1^ indicating acute toxicity, and 500–1400 μg∙L^−1^ indicating chronic toxicity [23,24]. The median lethal dose of various selenium compounds ranges between 1.5 and 6 mg∙kg^−1^ of body weight [15].

A characteristic sign of acute selenium poisoning is a garlic-like odor due to the pulmonary excretion of dimethyl selenide, vomiting, and tetanic spasm. Severe poisoning leads to death from respiratory failure [23,25]. Symptoms of chronic toxicity involve loss of hair and nails, lesions of the skin, and impairment in the nervous system [26]. 

### 2.2. Iodine in the Human Body

Iodine is the major constituent of the thyroid hormones thyroxine (T_4_) and triiodothyronine (T_3_). The recommended daily intake for an adult is approximately 150 µg of iodine per day for men, regardless of their age and physical activity. The recommended daily intake for women is 200 µg per day, for pregnant women, 250 µg, and for breastfeeding women, 300 µg [2]. The whole body of an adult contains approximately 20 mg of iodine, of which up to 80% is found in the thyroid gland. Another significant reservoir of iodine in the human body is found in the salivary glands [27]. Approximately 115 μg of iodide is taken up by the thyroid gland daily, of which approximately 75 μg is used for hormone synthesis and thyroglobulin storage, with the remainder returned to the extracellular fluid. The thyroid gland stores approximately 10 mg as a supply to protect the body during periods of iodine deficiency. Iodine is excreted by urine; thus, urinary iodine content is the most appropriate indicator of its intake [28].

The major impact of iodine deficiency is hypothyroidism, which results in impaired neurodevelopment, particularly in early life. The offspring of deficient mothers are at high risk of developing cognitive disability. In severe cases, iodine deficiency can lead to miscarriage and stillbirth [6]. In children, insufficient mental development due to iodine deficiency can cause cretinism, dementia, and delayed mental development. Hypothyroidism and goiter can develop in all ages. Endemic cretinism is often characteristic of iodine-deficient areas [29].

Similar to deficiency, excessive iodine intake can also be dangerous. Iodine-induced hyperthyroidism is related to increased iodine intake and occurs in populations that already have a severe iodine deficiency. After exposure to high iodine levels, a normal thyroid gland responds with an acute reduction in the synthesis of thyroid hormones called the Wolff–Chaikoff effect [30]. Long-term iodine exposure leads to the increased iodination of thyroglobulin, which increases its antigenicity and initiates the autoimmune process in genetically susceptible individuals, of which Hashimoto’s thyroiditis represents the most frequent form leading to the total destruction of the thyroid gland [31]. Graves’ hyperthyroidism is caused by autoantibodies to the thyroid-stimulating hormone receptor [32] and induces excessive thyroid hormone secretion.

### 2.3. Effect of Interactions of Iodine and Selenium on Thyroid Health

While iodine is a constituent of the two major thyroid hormones, selenium is incorporated in selenocysteine, which contains glutathione peroxidases, these playing an important role in thyroid gland protection [1]. A low selenium concentration was associated with a larger thyroid volume [33]. Selenium is beneficial in the treatment of autoimmune thyroid disorders. In patients with Hashimoto’s disease, selenoproteins reduce local inflammatory reactions, thus inhibiting the formation of TPO antibodies. This results in the improvement of the ultrasound structure of the thyroid gland. In Graves’ disease, the administration of Se can contribute to the promotion of euthyroid. Furthermore, among pregnant women, the administration of selenium decreased the occurrence of postpartum thyroiditis and definitive hypothyroidism in women positively tested for TPO antibodies [34]. Due to the close interaction of Se and I in thyroid metabolism and health, it is assumed that Se deficiency may endanger the effectiveness of salt iodization programs [35].

Goitrogens are compounds that can alter thyroid morphology and function by acting directly on the gland or by affecting its regulatory mechanisms. These include contaminants such as polyphenols and phenol derivates, pyridines, phthalate esters, polychlorinated and polybrominated biphenyls (PCB and PBB), and polycyclic aromatic hydrocarbons (PAH) [36]. In addition, many goitrogens occur in edible plants; such compounds include thiocyanate (CSN^−^), which can be found in plants from the Brassicaceae family; disulfides, which are a major component of onion and garlic odor; and flavonoids [36,37]. Inorganic compounds such as lithium and even excess iodine can act as a goitrogen. Prescription lithium-carbonate is the main cause of drug-induced hypothyroidism, while the iodine-rich drug amiodarone, prescribed for the treatment of cardiac arrhythmias, was reported to induce hyperthyroidism in 10% of patients [36].

### 2.4. Importance of Iodine and Selenium for Livestock

In cattle, selenium deficiency is linked to oxidative stress and tissue degeneration, such as White Muscle Disease (WMD). Even in cases where the clinical manifestation of WMD is absent, less severe Se deficiency may be linked to newborn calves experiencing a lack of vigor or thrift, commonly referred to as Weak Calf Syndrome. Other clinical conditions commonly associated with selenium deficiency include retained placenta and reductions in immune competence, particularly the function of phagocytic cells [38]. Liver damage in pigs was also associated with selenium deficiency [39].

Iodine deficiency is rare in modern production systems, however—primarily due to the fortification of salt, which has had a dramatic impact on helping to meet the iodine requirements of both livestock and humans [38]. Deficiency disorders in animals have a very similar character to human disorders. For animals, iodine intake is strongly influenced by the presence of goitrogenic substances in the diet, impairing I uptake by the thyroid [38,40]; thus, for example, feeding kale to farm animals is not recommended [40]. Simultaneous Se and I deficiency is likely to impair the ability of neonatal animals to generate heat and could have implications for their survival during cold stress [41].

## 3. Selenium and Iodine in Foodstuffs

The highest values of both selenium and iodine are found in protein-rich foods, such as fish, milk, meat, and eggs, while vegetables provide the lowest values [42,43,44,45]. Major food sources of Se include cereal-based foods and meat (26% of RDI), while the rest of the Se requirement is met by milk/beverages (21% of RDI), seafood/fish (10% of RDI), vegetables and fruits (7% of RDI), eggs (4% of RDI), and others [46,47]. Although the Se content of vegetables is low, lentils are an excellent source of essential micronutrients, including Se; a 50 g serving contains about 30 µg of Se [48]. The main sources of iodine in the diet are seafood if available; in inland areas, the main sources are milk, meat, eggs, dairy products, and to a lesser extent plants [6,49]. Since seaweed accumulates the highest amount of iodine, excessive seaweed consumption was found to be associated with goiter, hypothyroidism, and Hashimoto’s thyroiditis in countries where marine algae are traditionally consumed [50]. The iodine intake of the population in Japan, where a seaweed diet is common, is estimated at 1–3 mg∙day^−1^ [51]. Average values of the selenium and iodine contents of selected foods are represented in Table 1. 

Several countries have made various efforts to eliminate selenium and iodine deficiency, and many of them have been successfully implemented; however, deficiency disorders persist in some areas mainly due to a lack of legislation or knowledge, nutrient-deficient arable soils, and misperceptions that fortification alters food taste or color [52,53]. The average human dietary intake of selenium has doubled in Finland since 1985, when the supplementation of multi-nutrient agronomic fertilizers with sodium selenate became compulsory [54].

**Table 1:** Iodine and selenium content in selected foodstuff.

Food	Iodine Content	Reference	Selenium Content	Reference
Milk	194.8 µg∙kg^−1^	[43]	12.5 µg∙kg^−1^	[42]
Cheese	77.1 µg∙kg^−1^	[43]	23.2 µg∙kg^−1^	[42]
Yoghurt	80 µg∙kg^−1^	[44]	12.4 µg∙kg^−1^	[42]
Butter	30.3 µg∙kg^−1^	[43]	24.0 µg∙kg^−1^	[42]
Wheat	74.4 µg∙kg^−1^	[43]	11.9 µg∙kg^−1^	[42]
Rice	143.2 µg∙kg^−1^	[43]	26.5 µg∙kg^−1^	[55]
Chicken meat	69–160 µg∙kg^−1^	[45]	144.6 µg∙kg^−1^	[55]
Beef meat	62–265 µg∙kg^−1^	[45]	145.5 µg∙kg^−1^	[55]
Marine fish	2453.4 µg∙kg^−1^	[43]	616.8 µg∙kg^−1^	[55]
Eggs	378.8 µg∙kg^−1^	[43]	240.1 µg∙kg^−1^	[55]
Bread	35.4 µg∙kg^−1^	[43]	22.3 µg∙kg^−1^	[55]
Potatoes	20 µg∙kg^−1^	[44]	1.5 µg∙kg^−1^	[42]
Beans	186.7 µg∙kg^−1^	[43]	152.6 µg∙kg^−1^	[42]
Cabbage	123.1 µg∙kg^−1^	[43]	13.7 µg∙kg^−1^	[55]
Tomatoes	65.1 µg∙kg^−1^	[43]	1.1–29.1 µg∙kg^−1^	[42]
Herbs and spices	160.1 µg∙kg^−1^	[43]	17–132 µg∙kg^−1^	[56]
Lettuce	455.5 µg∙kg^−1^	[43]	0.3–20 µg∙kg^−1^	[42]
Cucumber	194.8 µg∙kg^−1^	[43]	14.7 µg∙kg^−1^	[42]

Salt iodization has been implemented worldwide to battle iodine deficiency disorders [57]. In some countries such as Denmark, the use of fortified salt has been mandatory since 2000 [58]. However, potential iodine losses from iodized salt must be taken into account. About 20% of iodine is lost from salt during storage and another 20% during cooking [59]. Various cooking procedures reduce the iodine content of food prepared with fortified salt; major losses were reported from boiling (−40%), microwave cooking (−27%), roasting and frying (−10%) [60].

## 4. Selenium and Iodine in Plants

The first step in mineral nutrient uptake by plants is uptake by the roots followed by xylem transport, which is determined by the mineral transporters present, the release of enzymes (exudates) by the roots, and the release of organic acids by microorganisms found in the rhizosphere, as well as by soil nutrient availability. In xylem, nutrients are moved upwards by means of transpiration forces to the leaves, which act at that point as a “sink” for nutrients and carbohydrates. When the carbon accumulated by photosynthesis exceeds the requirements for respiration and growth, the role of leaves changes from that of a sink to that of a source, meaning that nutrients are transported by the phloem to fruits [11]. While Se is readily transported by the phloem, iodine transport is weak [61]; however, there are several studies suggesting the translocation of exogenous iodine into fruits [62,63,64,65]. 

Selenate and its organic compounds are bioavailable forms and are actively taken up via the sulphate pathway by sulphate transporters [66]. The chemical and physical resemblance between selenium (Se) and sulfur (S) establishes that both these elements share common metabolic pathways in plants. Both S and Se are part of the VI-A chalcogen group of elements. These elements compete in biochemical processes that affect uptake, translocation, and assimilation throughout plant development [66]. White et al. [67] suggested four possible interactions between Se and S based on their study on *Arabidopsis thaliana* L.: (i) Se and S enter the plant through multiple transport pathways with sulfate/selenate selectivities; (ii) sulfate in the rhizosphere inhibits selenate uptake; (iii) selenate in the rhizosphere promotes sulfate uptake possibly by preventing a reduction in the abundance/activity of sulfate transporters; and (iv) the existence of competition between Se and S for biochemical processes such as assimilation into essential proteins.

Once accumulated, Se is found in plants in various forms. In brown rice, over 70% of accumulated selenium was present in the form of organoselenium [68]. Protein-bound selenomethionine (SeMet) was observed as the predominant form of selenium storage in rapeseed [69]; the formation of Se-methyl-selenocysteine was observed in radish [70]. Pickering et al. [71] stated that young leaves and roots contain organoselenium almost exclusively, which indicates that the ability to biotransform selenium is developmentally specific; thus, mature leaf tissues contain more selenium than young ones.

Although it has been noted that the higher plants do not require selenium supplementation, experiments on fertigating soils with sodium selenate fertilizers showed that selenium supplementation does not only positively affect the food chain but also plant yield [72]. Even though there is no evidence of Se essentiality in non-accumulator plants, observations reported an increase in biomass production in accumulator plants endemic to seleniferous soils [73]. Some plant species of *Astragalus*, *Stanleya*, *Morinda*, *Neptunia*, and *Xylorhyza* can hyperaccumulate selenium in their shoots when they grow on seleniferous soils [74]. Selenium hyperaccumulators have enhanced expressions of S transport and assimilation genes and may possess transporters with higher specificity for selenate over sulfate [75]. According to Wang et al. [76], Se-methyl-selenocysteine contributes to selenium tolerance in selenium accumulating plants; thus, the methylation of selenocysteine prevents its incorporation into proteins. This was observed in hyperaccumulator *Astragalus bisulcate*, where in this process, S-methylmethionine is the methyl donor for selenocysteine methyltransferase [77].

Each plant has a maximum tolerable dose of Se, above which phytotoxic effects appear [70]. The high addition of Se to plants is toxic and may trigger pro-oxidative reactions [72,78]. Selenium toxicity can result in the stunting of growth, yield reduction, a decrease in the activity of plant enzymes, chlorosis, the withering and drying of leaves, decreased protein synthesis, and premature death of the plant [70,74]. Generally, selenite is considered more toxic than selenate due to its fast conversion to selenoamino acids, which are incorporated into plant proteins as a replacement for S; selenocysteine and selenomethionine are incorporated in the place of cysteine and methionine [74,79].

A beneficial effect of selenium on plants was observed in the paddy fields of southern China, where untreated industrial water is directly discharged into water bodies and then used for irrigation causing metal contamination. The combined application of selenium with silicate nanoparticles (Se_3_Si_2_) decreased cadmium and lead (Pb) in rice [68]. In small doses, Se can activate enzymes in the antioxidant system of plants [80]. Some studies have indicated the beneficial effects of Se with respect to mitigating physiological stress through improving tolerance levels [75], e.g., increasing crop production [70,81], crop quality, metal translocation [68], and seed production [82]. Hegedüsová et al. [83] observed an increase in photosynthetic pigment synthesis in pea after the foliar application of Se. It is assumed that selenium can activate protective mechanisms, which can alleviate stress in the chloroplast [84].

According to Hartikainen [72], plants act as a buffer against selenium overaccumulation in food chain; their growth is reduced at high selenium levels, thus they tend to synthesize volatile compounds to reduce excess selenium. Se volatilization in various plants has been observed; however, the mechanism is not clear and is dependent on the applied Se species. Most occurs probably due to microbial activity in the rhizosphere, which was demonstrated in broccoli plants: Se volatilization was reduced by 95% when broccoli roots were treated with the antibiotics penicillin-G and chlortetracycline [85]. Moreover, the rate of Se volatilization in fortified plants is strongly correlated with the Se concentration in the plant tissues [86] and also depends on the applied Se species [87].

Iodine uptake in plants is facilitated by H^+^/anion symporters, specific Cl^−^ channels, and H^+^/anion antiporters of root cells following the chloride pathway [61,88]. Some organic acid transporters facilitating halide transport may also be involved [89]. Iodine is taken up by plants as iodide, which is included in the metabolic process of the synthesis of iodosalicylates, iodobenzoates, iodotyrosine (I-Tyr), and plant-derived thyroid hormone analogues [90].

Several studies reported iodine toxicity in plants [91,92,93,94]. It is responsible for the so-called “Reclamation Akagare” disease in rice first described in paddy fields in Japan, this characterized by chlorosis and necrotic spots on the leaves [95]. However, a recent study on *Arabidopsis thaliana* L. showed that iodine in micromolar concentrations is beneficial for biomass accumulation and leads to early flowering. It is incorporated in plant cells as iodinated proteins in the chloroplast and is functionally involved in the photosynthesis process; furthermore, it also regulates the expression of genes involved in stress defense mechanisms [96]. It is assumed that iodine improves the response to salinity stress by (i) preventing the uptake of Na^+^ and Cl^−^ by plants, (ii) the activation of antioxidant enzymes, and (iii) increasing the concentration of soluble sugars (sucrose, glucose, and fructose), which participate as osmoprotectants during osmotic adjustment [97].

Despite the possibility of iodine toxicity to plants, it is reported that low concentrations of iodine increase yield, biomass production, and plant qualitative parameters [62,63,64,97,98]. Some studies indicated that the detrimental effect of iodate is not as severe as that of iodide since iodate is not as readily bioavailable as iodide and its reduction is probably needed prior to absorption [99,100,101]. The beneficial effects of selenium and iodine on plant growth was recently reviewed by Golubkina et al. [102].

Plants have a specific mechanism for volatilizing accumulated iodine, this compromising the success of biofortification processes. So far, the production of methyl iodide has been observed in several species from 44 families and 33 orders [103]. Therefore, accumulated iodine is preferentially volatilized as methyl-iodide, this through the activity of specific halide/bisulfite methyl transferase catalyzing the S-adenosyl-L-methionine-dependent methylation of halides [104] encoded by *HOL* (*Harmless to Ozone Layer*) genes [105]. There are two types of *HOL* genes, of which *HOL-1* is responsible for iodine methylation [106].

Although iodine can be accumulated in plants, no known terrestrial plants are yet known as iodine hyperaccumulators.

## 5. Selenium and Iodine Biofortification Strategies

Although this paper focuses on the biofortification of plants, remarkable progress in meat biofortification is also worth mentioning. Biofortified meat products can contribute significantly to the reduction of mineral deficiency in humans.

### 5.1. Biofortification of Meat

Developing tailor-made fortified farmed fish is a promising means of increasing mineral intake. The regular consumption of seafood and fish products is highly recommended, as these products improve human health and help to prevent chronic pathologies such as cardiovascular diseases, diabetes, and obesity [107].

Supplementing the diets of gilthead seabream (*Sparus aurata*) and common carp (*Cyprinus carpio*) with iodine-rich seaweed and selenized yeast resulted in increased iodine, selenium, and iron contents in fish meat and fish oil, and lower contents of Hg and Cd in fish oil. The consumption of 150 g of fortified seabream made significantly higher contributions to the recommended daily intake of iodine (10%) and selenium (76%) compared to non-fortified fish [108]. 

A study investigating the effect of the addition of dried sugar kelp (*Saccharina latissima*) showed that adding 2% kelp to feeds of rainbow trout (*Oncorhynchus mykiss*) significantly increased the iodine content of fillet without increasing As, Cd, Hg, and Pb levels. The consumption of 160 g of fortified fillet provided 60% of the recommended daily intake of iodine and 50% of the recommended daily intake of vitamin D for adults [109].

The supplementation of farm animal diets with seaweed also has potential with respect to meat biofortification. The inclusion of 5% seaweed in the finishing lamb diet increased both Se and I contents in raw meat and I contents in dry-cured leg, resulting in an iodine concentration of 130 µg∙100 g^−1^, which corresponds to 60% of RDI [110]. Se-enriched probiotics were added to the corn-soybean diet of chicken. This enrichment significantly increased the physical and sensory characteristics of breast meat [111]. Se supplementation also slightly increased body weight and both Se and vitamin E content in chicken meat [112].

### 5.2. Plant Biofortification Strategies

Mineral nutrients must be effectively taken up from the substrate and deposited in plant tissues. Minerals are often unevenly distributed among different plant parts, though for a successful biofortification process, plants should accumulate minerals in their edible parts [11]. Still, scientists consider the bioaccessibility of micronutrients a more important and challenging issue [9]. Currently, there are three main strategies which have been successfully applied to improve the nutritional contents of crops. These are (i) agronomic biofortification, (ii) conventional breeding, and (iii) genetic engineering [11].

#### 5.2.1. Agronomic Biofortification 

Agronomic biofortification is considered a relatively simple method with immediate results [11]. This approach is mainly focused on optimizing the application rate of mineral or organic fertilizers containing the desired nutrient or the improvement of the mobility and bioavailability of mineral nutrients already present in the soil [61]. The regular application of fertilizers is needed, which may be expensive and carries the possibility of negative environmental impacts and the exhaustion of reserves [11]. According to White and Broadley [61], selenium reserves are expected to be exhausted in less than 40 years when applied regularly.

Mineral elements in soils can be present as free ions, as surface-adsorbed ions, in dissolved states, and as precipitates [61]. The low natural contents of minerals in soil limit their bioaccumulation rates, though limitations occur more commonly due to their low bioavailability to plants resulting from their interaction with soil particles and other biological processes [113,114]. Thus, the agronomic approach promotes the application of mineral fertilizers and the improvement of their solubilization in the soil [11]. The application of fertilizers as foliar sprays is an effective alternative to the agronomic approach in situations where mineral elements are not readily translocated to edible tissues [11]. Idrees et al. [115] suggested that seed priming followed by foliar spray is more economical and shows a higher net return compared with the foliar application only. Generally, the best way to enrich plants with selenium is foliar fertilization with selenate in increased doses [116]. The foliar application of selenium fertilizers such as Na_2_SeO_4_ and K_2_SeO_4_ is very effective and has an immediate effect on plant selenium concentration; less soluble fertilizers such as BaSeO_4_ and selenite are known to have a delayed effect but last for a longer time [117]. KIO_3_ added to irrigation water also showed prolonged effect [118].

To increase nutrient concentrations in the edible parts of selected crops without a negative impact on crop yield and physiological parameters, a toxicity assessment of the selected minerals should be first conducted. Furthermore, mineral species with lower toxicity and better transport in plant tissues should be selected [94]. The bioavailability of different chemical species of a mineral can differ among plant species. Better selenium accumulation was observed when Se was applied as selenate (VI) for potato [80], carrot [119], common buckwheat [120], wheat [121], lettuce [78], and turnip [122], while selenite (IV) was more effective in the biofortification of rice [123]. Recently, fertilizer products on a nanoscale level have been viewed as potential agro-inputs for precise micronutrient management, even at very low application rates—Se nanoparticles, for example, resulting in a higher growth-promoting effect than selenite or selenate [124,125,126]. Selenomethionine [126] and Se with chitosan–polyacrylic acid complex [127] also exhibited a higher transfer coefficient among selenium species and a substantial positive effect on growth parameters and grain yield with respect to wheat and maize and lettuce.

Special organic fertilizers may also be applied to enhance Se and I contents in crops. Plants used for Se phytoremediation can be used as organic Se fertilizers. Powdered *Stanleya pinnata* was successfully used to enrich broccoli and carrots with Se [128,129]. *Astragalus bisulcatus* extract with a high methyl-selenocysteine content was used to biofortify buckwheat with selenium [130], and iodine-rich seaweed fertilizers were successfully applied to enhance the iodine content of crops [131,132].

Among applied iodine species, iodide is considered more mobile; higher accumulation rates were observed in carrot [133], common buckwheat [120], and barley [100]; however, its deleterious effects are more pronounced compared to iodate [94]. On the other hand, Lawson et al. [134] observed greater yield in lettuce and kohlrabi from foliar iodide application, while iodate-treated plants showed higher accumulation rates. Fertigation with 5-iodosalicylic acid resulted in higher I accumulation rates in lettuce compared to fertigation with KIO_3_ and 3,5-diiodosalicylic acid [135]

The presence of other elements in soils can also be a limiting factor in the accumulation of the selected mineral. In addition, plants may accumulate other elements which are not nutritionally feasible. Among the elements that favor selenium uptake is nitrogen, which is administered in fertilizers to intensify the yield. Reis et al. [136] reported that increasing the nitrogen dose in rice grains increased the selenium content and increased protein synthesis. The co-application of selenium with an N carrier improved the efficacy of Se fertilizers without additional application costs in the field [137]. Sulfur was reported to increase selenium uptake from organic-rich soils [69] and bulb yield in onion [138]; contrariwise, selenate was shown to promote the accumulation of S and P in buckwheat [130]. Another element influencing selenium intake is iodine; foliar application of iodine on Indian mustard caused a decrease in selenium uptake by 4% [124].

The application of nano-Si to chervil jointly biofortified with selenium and iodine was shown to enhance growth and the biofortification rate; in addition, a higher production of photosynthetic pigments and antioxidant activity, and thus a decrease in nitrate and sodium accumulation was observed [139].

The co-application of iodine with salicylic acid had a positive effect on iodine accumulation in potato [140], lettuce [141], and tomato [142], but no significant differences were observed in yield; the addition of humic acids positively affected iodine accumulation in spinach, but did not affect yield [143].

Agronomic biofortification has several limitations including sorptive interactions with soil components, which reduce the bioavailability of applied micronutrient fertilizers [100,101,144,145].

The low contents of minerals in plants are not always the result of their low contents in soils; more often it is due to their presence in forms that are unavailable for plants. Increased bioavailability from soils can be achieved by the use of various microorganisms, so-called bio-fertilizers [10,146]. However, this method cannot be applied for iodine biofortification, because iodine is biovolatilized as methyl iodide at high rates by both bacteria and microscopic filamentous fungi [100,147,148,149].

Yang et al. [150] reviewed the use of microorganisms in Se biofortification strategies, and concluded that the role of microorganisms in biofortification resides in (i) altering soil properties and impacting the redox chemistry of Se to improve bioavailability; (ii) regulating root morphology and stimulating the release of secretions facilitating Se uptake; (iii) upregulating the expression of genes and proteins related to Se metabolism in plants; and (iv) stimulating the generation of certain metabolites in plants contributing to Se absorption.

Selenium associated with soil-borne Se tolerant bacteria such as *Stenotrophomonas*, *Bacillus*, *Enterobacter*, and *Pseudomonas* can be translocated into plants [151]. Durán et al. [152] reported that inoculating lettuce with selenobacteria and arbuscular mycorrhizal fungi enhanced Se content in the plant and also promoted macro- and micronutrient uptake and improved its tolerance to drought stress. The inoculation of wheat seeds with *Bacillus pichinotyi* and *Bacillus licheniformis* increased Se and Fe accumulation in wheat [153,154]; the latter also enhanced S and Ca uptake [154].

#### 5.2.2. Conventional Plant Breeding

Conventional plant breeding along with genetic approaches are considered long-term biofortification strategies [10]. While traditional plant breeding focuses on increasing crop yield, resistance to pathogens, and tolerance to abiotic stresses, in biofortification strategies, greater emphasis is placed on enhancing micronutrient and vitamin concentrations in the edible parts of the plant [9,11]. This method takes advantage of the difference in uptake rates among cultivars [133,155] or even between the sexes of plants [124]. In studies on spinach, different degrees of enrichment were observed depending on the sex of the plant. Male plants accumulated selenium at higher rates than females; furthermore, in biofortified spinach, the nitrate content and accumulation of polyphenols and ascorbic acid were also higher in males, indicating the possible participation of phytohormones in the selenium accumulation process [156].

#### 5.2.3. Genetic Engineering

In cases when the desired trait is absent in the genotypic variation of the plant, genetic engineering can be applied to increase the mineral uptake of the desired micronutrient through the reconstruction of the accumulation pathways of accumulator plants. Advances in genetic engineering are similar to those in conventional plant breeding; it is a one-off cost method and can speed up the process of conventional plant breeding. On the other hand, development might take a long time. The biggest negative of this approach is the low public acceptance of genetically modified crops among both consumers and farmers; thus, organically grown crops are considered safer and more nutritious. The success of this approach, as in the case of conventional breeding, is limited to minerals already available in soils [11]. It was shown in *Arabidopsis thaliana* L. that iodine content in a plant can be increased by knocking out the *HOL-1* gene, thus decreasing the volatilization of accumulated iodine from plants [105,157], or by overexpressing the human sodium-iodide symporter for increased uptake [106].

## 6. Simultaneous Biofortification of Crops with Selenium and Iodine

According to Lyons [158], the potential benefits of applying selenium and iodine together, along with the economy of combining them with the application of strategic fertilizer and pesticide, could make biofortification commercially viable for farmers. In recent years, several studies have been conducted on the simultaneous agronomic biofortification of crops with iodine and selenium, often combined with zinc and iron, with promising results. 

A widespread study on the simultaneous biofortification of wheat with I, Se, Fe, and Zn was presented by Zou et al. [159]. Biofortified wheat was grown for over 2 years at 27 field locations in China, India, Mexico, Pakistan, South Africa, and Turkey. The authors observed an increase in iodine concentration from 24 to 249 µg∙kg^−1^ in grains. The selenium concentration increased from 90 to 338 µg∙kg^−1^ without yield loss in all participating countries. The micronutrient cocktail that was applied in an aqueous solution, the dose ranging between 600 and 800 L·ha^−1^, contained 0.5% ZnSO_4_·7H_2_O + 0.05% KIO_3_ + 0.2% FeEDTA + 0.001% NaSeO_4_ *w*/*v* and was applied twice: the first spray was applied one week before heading and the second spray at the early milk stage. The same micronutrient cocktail was used to successfully increase the concentration of all four micronutrients in wheat [159,160]. However, Cakmak et al. [160] reported that a foliar application of iodine during the growth period resulted in higher iodine content in wheat. The co-application of I, Se, Fe, and Zn resulted in lower iodine accumulation in plant tissue compared to plants subject to the application of iodine alone. Prom-u-thai et al. [161] found that the foliar application of I and a cocktail of Zn + I + Fe + Se both enhanced I concentration in grain—from 11 to 204 µg kg^−1^ with the application of I alone, and to 181 µg kg^−1^ with the cocktail. The grain concentration of Se was also elevated via the micronutrient spray and increased from 95 to 380 mg kg^−1^, while fertigation did not have a significant effect on the yields of rice grain.

Germ et al. [162] investigated the effects of several species of selenium and iodine and their combinations on the biofortification efficiency of chicory. The selenium content in Se-treated plants was increased by up to 5.5-times, while the iodine content was 3.5 times greater than in control plants. Iodide or iodate applied together with selenite in the spray solution increased the uptake of Se, while both forms of iodine, applied together with selenate, reduced the uptake of Se. The different treatments had no significant effects on the yield and mass of the chicory heads, but plants treated with iodate had lower amounts of chlorophyll *a* and carotenoids than the control.

In potato, the addition of iodine, selenium, and salicylic acid to nutrient solutions did not cause any significant changes in the total number or mass of potato tubers; thus, their increased concentration was observed. The application increased the contents of N, K, and Na in tubers and decreased the content of Mn and Zn in roots [140].

Smoleń et al. [163] conducted a three-year study to examine the effect of the combined application of selenium (Na_2_SeO_4_, 0.25 kg Se ha^−1^) and iodine (KI, 4 kg I ha^−1^) in carrots. The yields of the carrot plants were not affected by the combined biofortification, and the biomass and share of marketable yield of biofortified plants were similar to those of the control. However, compared to the control, the I and Se contents in roots of biofortified plants increased 7.7 and 4.9-times, respectively. The authors state that the intake of 100 g of biofortified carrot would substantially cover the recommended daily intake for I and Se. Soil fertilization with I and Se did not significantly alter the nutritional quality of carrot roots; however, the co-application of iodine with selenium enhanced iodine enrichment. Another study on carrots [90] also showed that there was no statistically significant effect on the quantitative and qualitative parameters of carrots, when I, Se, or I + Se were applied via foliar biofortification. Previously, Smoleń et al. [133] reported higher Se and I accumulation in carrot when I was applied as iodide.

The I-Se-Zn biofortified soilless-grown lettuce significantly increased concentrations of Se and Zn in leaves but I concentrations did not improve with the application of these elements. Combined biofortification with I-Se-Zn had no significant effect on the concentrations of the majority of nutrients (P, K, Ca, Mg, Na, Al, Cl, Mo, Cu, Cd, Rb, Sr, Cr, Zr, Nb, Ta, Th, Ga, Sn, Sb), whereas S, Si, Ba, La, Pb, Bi, and Ti increased and Fe, Mn, Co, Ni, and Br decreased [164]. 

Another study showed that the application of Na_2_SeO_4_ (both alone and with KI or KIO_3_) led to a decrease in lettuce yield and an increase in dry matter content in lettuce; furthermore, each year, the content of dry matter was higher in lettuce grown on fertilized soil [165].

Golubkina et al. [124] observed synergistic effects on the part of Se and I in inhibiting nitrate accumulation in Indian mustard enhanced flavonoid biosynthesis and in decreasing Cd and Sr accumulation.

In pumpkin, the accumulation of Se was higher in sprouts from seeds treated with Se together with I, compared to sprouts from seeds treated with Se alone. Pumpkin yield and seed production were unaffected [166].

The spraying of I-containing solutions on apple and pear trees resulted in leaf necrosis, the intensity of these symptoms increasing as the number of applications increased and the growing season progressed. The co-application of KNO_3_ and Na_2_SeO_4_ with I did not affect the damage pattern [167]. More examples of the co-application of selenium and iodine are shown in Table 2.

## 7. Content and Stability of Selenium and Iodine in Biofortified Plants

According to several studies, biofortified plant products have been proven to be an effective source of nutrients. According to Tonacchera et al. [168] and Jiang et al. [118], the consumption of biofortified vegetables significantly increases urinary iodine concentration. The coverage of 100% of the recommended daily intake of iodine was reported in single servings of fortified tomato [62], pepper [64], and strawberries [63]. Golubkina et al. [124] reported that the consumption of 100 g of mustard leaves fortified with 50 mg∙L^−1^ sodium selenate and 100 mg∙L^−1^ potassium iodide applied via foliar spray could provide 100% and 15% of the recommended daily intake of selenium and iodine, respectively. According to Smoleń et al. [133], the consumption of 100 g of carrots fortified with 1 kg∙ha^−1^ selenium and 5 kg∙ha^−1^ iodine can supply 100% of the recommended daily intake for both nutrients. In another study, the authors stated that the consumption of biofortified onions treated with 25 µM selenium would exceed the recommended daily intake of selenium in countries where the daily average consumption of onions exceeds 24 g [138].

The simultaneous agronomic biofortification with selenium and iodine needs to be further optimized, since coapplying I and Se in fortifier cocktails results in a lower concentration of iodine in grains, as well as a too-high concentration of selenium in grains when applied in the doses reported by Cakmak et al. [160] for use in regions with high bread consumption. However, they stated that a single application of the same amount of iodine three-times during the growth period resulted in a bread iodine content which exceeded 100% of RDI on the basis of a 400 g serving of bread, which is also not recommended in these countries.

For a biofortification process to be successful, micronutrients must not only exhibit enhanced levels in edible tissue, but must also be bioavailable for absorption in the human gut [11]. Studies on the bioaccessibility of selenium and iodine from fortified plants are promising. Iodine was shown to be sufficiently bioaccessible in the gut during the simulated digestion of bread and cookies made from biofortified wheat [160]. The bioaccessibility of iodine from biofortified celery after digestion in simulated gastric and intestinal juice amounted to 74% and 68%, respectively [169]. Sun et al. [170] reported that, after cooking, up to 76% of the total selenium content in biofortified rice was bioaccessible.

**Table 2:** Concentrations of I and Se in biofortified plants.

Species	Application	I Dose	Se Dose	Control		I Accumulated	Se Accumulated	Reference
				I	Se			
Carrot	Soil spray	4000 g·ha^−1^ (I^−^)	250 g·ha^−1^ (SeO_4_^2−^)	73.5 µg·kg^−1^	220.5 µg·kg^−1^	546 µg·kg^−1^	1315 µg·kg^−1^	[163]
	Foliar spray	400 g·ha^−1^ (I^−^)	20 g·ha^−1^ (SeO_4_^2−^)	28.5 µg·kg^−1^	11.3 µg·kg^−1^	567 µg·kg^−1^	198.4 µg·kg^−1^	[90]
Rice	Soil and foliar spray	0.05% (IO_3_^−^)	0.001% (SeO_4_^2−^)	11 µg·kg^−1^	65 µg·kg^−1^	25-355 µg·kg^−1^ *	90-602 µg·kg^−1^ *	[161]
Radish	hydroponic	1 mg·L^−1^ (IO_3_^−^)	1 mg·L^-1^ Se(VI)	15 µg·g^−1^	13 µg·g^−1^	292.1 µg·g^−1^	312.6 µg·g^−1^	[171]
Cabbage	Foliar spray	1 g·L^−1^ (I^−^)	10 mg·L^-1^ Se(VI)	0.065 µg·g^−1^	0.030 µg·g^−1^	0.286 µg·g^−1^	0.578 µg·g^−1^	[172]
Lettuce	Irrigation drainage	150 µM (IO_3_^−^)	20 µM (SeO_4_^2−^)	2.42 mg·kg^−1^	0.7 mg·kg^−1^	2.47 mg·kg^−1^	104 mg·kg^−1^	[164]
	Floating system	5 µM (I^−^)	13 µM (SeO_4_^2−^)	4.7 mg·kg^−1^	0.09 mg·kg^−1^	72.55 mg·kg^−1^	3.44 mg·kg^−1^	[173]
	Aeroponic system	5 µM (I^−^)	13 µM (SeO_4_^2−^)	4.02 mg·kg^−1^	0.1 mg·kg^−1^	71.9 mg·kg^−1^	6.46 mg·kg^−1^	
	Hydroponic	30 µg·L^−1^	8.5 µg·L^−1^	3.5 mg·kg^−1^	1.5 mg·kg^−1^	254.1 mg·kg^−1^	9.4 mg·kg^−1^	[165]
Apple	Foliar spray	2 × 1.5 kg IO_3_^−^ (ha·m CH **)^−1^	2 × 0.05 kg SeO_4_^2^ (ha·m CH)^−1^	0.76 µg·100 g^−1^	0.4 µg·100g^−1^	43.3 µg·100 g^−1^	2.7 µg·100 g^−1^	[167]
Pear	Foliar spray	3 × 1.5 kg IO_3_^−^ (ha·m CH)^−1^	3 × 0.05 kg SeO_4_^2^ (ha·m CH)^−1^	1.1 µg·100 g^−1^	0.1 µg·100g^−1^	59.9 µg·100 g^−1^	2.1 µg·100 g^−1^	
Pumpkin seeds	Seed soaking	1000 mg·L^−1^ (IO_3_^−^)	10 mg·L^−1^ (SeO_4_^2−^)	30 ng·g^−1^	35 ng·g^−1^	20 ng·g^−1^	87 ng·g^−1^	[166]
Chicory	Foliar spray	1000 mg·L^−1^ (IO_3_^−^)	10 mg·L^−1^ (SeO_4_^2−^)	20.2 ng·g^−1^	14.2 ng·g^−1^	79.3 ng·g^−1^	71 ng·g^−1^	[162]
Potato	Hydroponic	30 µg·L^−1^ (IO_3_^−^)	8.5 µg·L^−1^ (SeO_3_^−^)	118.5 µg·kg^−1^	255.75 µg·kg^−1^	831 µg·kg^−1^	2695 µg·kg^−1^	[140]

* depending on soil type; ** canopy height.

The stability of nutrients in ingredients made from fortified plants is also important, especially for iodine because of its volatile nature. As discussed earlier, a significant proportion of iodine is volatilized from iodized salt during cooking and various cooking procedures; thus, boiling of fortified vegetables further reduces their iodine content. Nevertheless, iodine losses originating from iodized salt during cooking procedures are higher than from biofortified vegetables [169]. The boiling of biofortified rice resulted in a 40% decrease in iodine content compared to the original grains [160]. Slightly lower iodine losses from biofortified celery, amounting to 15%, were observed after cooking biofortified celery [169]; however, no significant iodine loss was detected during the cooking of fortified potatoes [174] nor for tomato after cooking if the peel was retained [62]. Losses during cooking most likely occur due to iodine leaching into the boiling water [160]. Selenium during boiling is more stable; Sun et al. [170] reported that boiling at low rice/water ratios did not affect the selenium content of biofortified rice.

In contrast to boiling, baking did not affect iodine content in bread or focaccia made from biofortified wheat flour and potatoes, respectively [160,174]; however, Cerretani et al. [174] observed a slight decrease in the iodine content of vegetable pie made from biofortified potatoes.

## 8. Conclusions

Biofortification intervention is a promising crop-based strategy for eliminating micronutrient malnutrition. By using the right chemical forms and by employing the right application methods, plant food biofortification can be a cost-effective approach to increasing the daily selenium and iodine intake. After setting target nutrient levels and identifying the most suitable strategies for target crops, changes in the levels of minerals due to crop processing (such as the removal of outer tissues while peeling), food preservation (drying, canning), cooking procedures (boiling, frying, baking), and storage should be investigated. Such effects have not yet been determined for the majority of biofortified crops. Such studies focusing on the effect of food processing could provide essential information with respect to optimizing fertilizer micronutrient contents and improving the methodologies of biofortification processes.

In the future, the consequences of global warming and population growth will cause a global decrease in the amount of arable land. Furthermore, mineral deficiencies are expected to become more salient. Thus, biofortification strategies could represent more sustainable tools for mitigating micronutrient malnutrition.

## Data Availability

Not applicable.
